# Towards a New Definition of Return-to-Work Outcomes in Common Mental Disorders from a Multi-Stakeholder Perspective

**DOI:** 10.1371/journal.pone.0039947

**Published:** 2012-06-29

**Authors:** Hiske L. Hees, Karen Nieuwenhuijsen, Maarten W. J. Koeter, Ute Bültmann, Aart H. Schene

**Affiliations:** 1 Program for Mood Disorders, Department of Psychiatry, Academic Medical Center, University of Amsterdam, Amsterdam, The Netherlands; 2 Coronel Institute of Occupational Health, Academic Medical Center, University of Amsterdam, Amsterdam, The Netherlands; 3 Department of Health Sciences, Community and Occupational Medicine, University Medical Center Groningen, University of Groningen, Groningen, The Netherlands; University of Western Brittany, France

## Abstract

**Objectives:**

To examine the perspectives of key stakeholders involved in the return-to-work (RTW) process regarding the definition of successful RTW outcome after sickness absence related to common mental disorders (CMD’s).

**Methods:**

A mixed-method design was used: First, we used qualitative methods (focus groups, interviews) to identify a broad range of criteria important for the definition of successful RTW (N = 57). Criteria were grouped into content-related clusters. Second, we used a quantitative approach (online questionnaire) to identify, among a larger stakeholder sample (N = 178), the clusters and criteria most important for successful RTW.

**Results:**

A total of 11 clusters, consisting of 52 unique criteria, were identified. In defining successful RTW, supervisors and occupational physicians regarded “Sustainability” and “At-work functioning” most important, while employees regarded “Sustainability,” “Job satisfaction,” “Work-home balance,” and “Mental Functioning” *most* important. Despite agreement on the importance of certain criteria, considerable differences among stakeholders were observed.

**Conclusions:**

Key stakeholders vary in the aspects and criteria they regard as important when defining successful RTW after CMD-related sickness absence. Current definitions of RTW outcomes used in scientific research may not accurately reflect these key stakeholder perspectives. Future studies should be more aware of the *perspective* from which they aim to evaluate the effectiveness of a RTW intervention, and define their RTW outcomes accordingly.

## Introduction

Common mental disorders (CMD’s), such as depression and anxiety disorders, are highly prevalent [1] and one of the leading causes of sickness absence in industrialized countries [2]. Sickness absence due to CMD’s has not only substantial negative effects for the employee, but also results in major costs for employers and society [3]. This high individual and societal burden has resulted in an increase of interventions that aim to promote return-to-work (RTW) after CMD-related sickness absence [4]–[13].

Although criteria for defining RTW vary according to the researcher’s discipline or socio-legal context [14], most researchers tend to use criteria that are easy to measure, such as work status (present/absent from work) [12], number of hours worked [11], [13], or time until an employee returns to work for the full number of contract hours [8], [15], [16] with equal earnings [6], [10]. Based on such criteria, it is decided whether a certain intervention is effective in promoting RTW, and consequently, whether this intervention is implemented by employers and/or funded by health insurers [17]. Although not (often) explicitly stated, it is implied that these criteria reflect a valid operationalization of “successful” RTW.

To our knowledge, however, no study in the mental health field has examined to what extent such criteria actually reflect what key stakeholders (e.g. employees, employers, occupational health professionals) find most important for successful RTW. Rather, previous studies have mainly focused on key stakeholder perspectives pertaining to the RTW *process* (i.e., what factors during the RTW process impede or facilitate an employees’ RTW?) [18]–[21].

A handful of studies in the physical health field have examined the *employee*’s perspective in defining successful RTW, suggesting that employees regarded their productivity, a sense of having done something meaningful [22], their job satisfaction, and the relationship with their supervisor [23], [24] as much more significant than the criteria traditionally used for evaluating RTW outcomes (e.g. hours worked or income earned). However, other key stakeholders such as supervisors or occupational physicians (OP’s) may apply different criteria, depending on their own interests [19]. For example, a supervisor may give priority to the number of hours worked. The OP, in turn, may find it most important that an employee returns to work in a job that fits with his current level of functioning.

Considering the marked increase in CMD-related sickness absence [25], it is vitally important to explore these different perspectives: If current definitions of successful RTW fail to include criteria most important to key stakeholders, current study conclusions (i.e., is the intervention effective?) may not accurately reflect their views [26]. This may hamper sustainable implementation [26], [27]. More knowledge regarding the various stakeholder perspectives may also guide the development of new RTW interventions.

Therefore, the goal of the present study is to identify key stakeholders’ perspectives on what constitutes successful RTW after CMD-related sickness absence. We used a mixed-method design [28], the sequential exploratory method [29], in which a qualitative study phase was followed by a quantitative study phase. In the qualitative study phase, we identified a broad range of criteria important to the definition of successful RTW from the perspectives of key stakeholders, and categorized these criteria into content-related clusters. In the quantitative study phase, we asked a larger sample of stakeholders to select the most important clusters and criteria for successful RTW. The methods and results section will be described separately per study phase.

## Study Phase 1: Identifying Important Criteria and Clusters for Successful RTW

The Medical Ethics Committee considered ethical approval not necessary. Written informed consent was obtained from all employees who participated in the focus groups and telephone interviews. Data from the online questionnaire were analyzed anonymously.

### Method

#### Participants

A convenience sample of employees, supervisors, OP’s and researchers was used. Employees were recruited through mental health professionals at three mental health institutions. Inclusion criteria were: diagnosis of depressive, anxiety, or adjustment disorder according to DSM-IV criteria [30]; history of sickness absence for at least four weeks; and have returned to work or currently in the RTW process. Employees were excluded if the primary reason for their sickness absence was a physical disease or a severe mental disorder (e.g., bipolar disorder, psychotic disorder).

OP’s and supervisors were recruited through conferences, professional associations, and a snowball approach (asking supervisors and OP’s to identify colleagues). Supervisors were included if they had experience with employees who had returned to work after a CMD-related sickness absence of at least four weeks. OP’s were selected if they had at least one year of experience with the RTW process of employees with a CMD-related sickness absence. To incorporate scientific knowledge during this broad examination of criteria, researchers within the work disability field were also included. Researchers who had published on RTW and CMD’s in peer-reviewed journals were approached by e-mail for participation.

### Procedure

#### Focus groups

Participants in each focus group were asked to write down all criteria they considered important for the definition of successful RTW on a brainstorm form. These criteria were then discussed among group members. The focus groups were coordinated by a facilitator (KN), while the co-facilitator (HH) took notes. To ensure that all criteria were included, participants handed in their individual brainstorm forms at the end of the session. After each focus group, notes were checked for accuracy using the audio-recorded material. All participants provided permission for the discussions to be audio-recorded.

#### Interviews

Because not all participants could be present for the focus groups, we also conducted semi-structured interviews by telephone with a format that resembled the focus groups as much as possible. Prior to the interview, participants received a description of the research project by e-mail, together with two attachments: 1) a brainstorm form, and 2) a summary form with the focus group results. Participants were asked 1) to write down all criteria they considered important for the definition of successful RTW on the brainstorm form, and 2) to look at the focus group results and to identify and add additional criteria. It was explicitly stated that agreement with the focus group results was not required, but that we aimed to identify a variety of criteria. During the interview, both forms were discussed, while notes were taken by the interviewer. After the interviews, notes were checked for accuracy using the audio-recorded material. All participants provided permission for the discussions to be audio-recorded.

### Data Analysis

HH and KN independently organized the criteria into content-related clusters. Naming of clusters and differences between raters regarding the allocation of criteria were compared in repeated discussions with the research team until consensus was reached. Criteria were excluded if they were unclear or unrelated to the main research question. If criteria were similar in content they were consolidated, rather than retained as separate criteria. Data were collected until saturation was achieved, i.e., when no new criteria emerged from the interviews.

### Results

Three separate focus groups (duration 95-120 minutes) were held with employees (N = 11), OP’s (N = 9), and researchers in the work disability field (N = 7). In addition, 30 telephone interviews (duration 14 − 35 minutes) were conducted with supervisors (N = 20), employees (N = 5), and OP’s (N = 5). After elimination of redundant criteria, a total of 52 unique criteria were identified. These criteria corresponded to 11 clusters: (1)‘Hours worked’, (2) ‘Work load’, (3) ‘Job function’, (4) ‘Income’, (5) ‘Sustainability’, (6) ‘Mental functioning’, (7) ‘At-work functioning’, (8) ‘Relationship with supervisor’, (9) ‘Relationship with colleagues’, (10) ‘Job satisfaction’, and (11) ‘Work-home balance’.

## Study Phase 2: Selecting the Most Important Clusters and Criteria for Successful RTW

### Method

#### Participants

Participants were recruited through various methods. Employees, supervisors, and OP’s who participated in study phase 1 were asked to also participate in study phase 2. Additionally, employees were recruited through mental health professionals at various mental health institutions. OP’s and supervisors were recruited through conferences and professional associations. In addition, about 5% of OP’s and supervisors were recruited through a snowball approach (i.e., asking supervisors and OP’s to identify colleagues). For each key stakeholder group, the same eligibility criteria were applied as in study phase 1.

#### Design and procedure

Based on the clusters and criteria identified in the first study phase, an online questionnaire (SurveyMonkey.com) was developed that aimed to identify the most important clusters and criteria. Prior to distribution of the questionnaire, a panel of different stakeholders (N = 11) critically assessed the items independently and submitted comments and revisions by e-mail. Next, participants received a link to the online questionnaire by e-mail. If the questionnaire was not filled out within 2 weeks, a reminder was sent. Participants who did not respond after two reminders were considered non-respondents. To preserve anonymity, data were de-identified by removing participants’ email addresses once the data were entered into SPSS. No names or other identifying information was asked through SurveyMonkey.

#### Questionnaire

The online questionnaire started with a short introduction to a generic case study (Mr. Janssen, an employee with CMD-related sickness absence who returned to work), which was used throughout the questionnaire to frame the questions. The completion of the questionnaire consisted of four steps: Participants were asked whether they considered a certain cluster important for successful RTW (step 1). If yes, participants selected the most important 1-3 criteria categorized under that cluster (step 2). After all clusters and criteria were reviewed, participants selected the clusters they found *most* important for evaluating whether Mr. Janssen had achieved successful RTW (step 3; max 3 clusters). The questionnaire concluded with a demographic section on gender, age, occupational field, company size, and years of work experience (step 4). The questionnaire consisted of 28 questions. For employees, two questions were added regarding their current percentage of work resumption and clinical diagnosis.

#### Data analysis

For each cluster, we calculated the percentage of employees, supervisors, and OP’s who selected that cluster among their top-three for successful RTW (in step 3). We also calculated the percentage of employees, supervisors, and OP’s who rated a certain criterion among their most important criteria (in step 2). Participants who rated a certain cluster ‘not important at all’ for successful RTW (in step 1) were excluded from these latter analyses of criteria.

A cluster was considered significant to a certain stakeholder group if ≥40% had selected this cluster among their top-three of most important clusters for successful RTW. A criterion was considered significant if ≥40% of a certain stakeholder group had selected this criterion among their most important criteria (within a cluster) for successful RTW. The 40% criterion was selected *a priori* by our research team, as we agreed that 40% reflected the *minimum* criterion for relevant agreement within a stakeholder group.

### Results

Of the 128 OP’s invited to complete the questionnaire, 80 agreed to participate, and 74 (58% of the invited) completed the questionnaire. Of the 85 supervisors invited to participate, 54 responded and 51 (60% of the invited) completed the questionnaire. Of the 65 invited employees, 58 responded and 53 (82% of the invited) completed the questionnaire. Only completed questionnaires were used in the analyses. Demographic characteristics are presented in Table 1.

**Table 1:** Demographic characteristics of employees (n = 53), supervisors (n = 51), and occupational physicians (n = 74).

	Employees	Supervisors	OP’s^a^
	(n = 53)	(n = 51)	(n = 74)
Age, years^b^	45(9)	48(9)	51(7)
Sex (% male)	47%	47%	51%
Sector			
Financial	36%	37%	36%
Healthcare	28%	33%	34%
Education	6%	18%	6%
Other	30%	12%	25%
Work experience, years^b^	22(10)	24(10)	23(7)
Company size			
1–15 employees	11%	6%	5%
16–50 employees	9%	10%	4%
51–150 employees	15%	9%	15%
>150 employees	64%	75%	76%
Mental disorder			
Mood disorder	64%		
Anxiety disorder	32%		
Adjustment disorder	4%		
Current percentage of work resumption			
1–50%	26%		
51–99%	26%		
100%	47%		

a OP  =  occupational physician. ^b^Mean (SD).

Employees considered job satisfaction (53%), mental functioning (51%), home-work balance (49%), and sustainability (40%) important (see Table 2). Supervisors and OP’s, in turn, regarded sustainability (supervisors  = 55%; OP’s  = 73%), and at-work functioning (supervisors  = 55%; OP’s  = 49%) important. Except for sustainability for OP’s (73%), only 40–55% selected a certain cluster among their top-three of most important clusters, indicating considerable heterogeneity within each stakeholder group.

**Table 2:** Percentage of participants who regarded the indicated cluster in their top-three of most important clusters for a successful RTW outcome (clusters are ordered according to their overall average percentage).^a^

Clusters	Employees	Supervisors	OP’s	Average
	(n = 53)	(n = 51)	(n = 74)^b^	(n = 178)^c^
Sustainability	**40%**	**55%**	**73%**	**56%**
At-work functioning	21%	**55%**	**49%**	**42%**
Work-home balance	**49%**	27%	38%	38%
Mental functioning	**51%**	27%	27%	35%
Job satisfaction	**53%**	23%	22%	33%
Relationship with supervisor	21%	35%	19%	25%
Work load	13%	29%	24%	22%
Number of hours worked	11%	20%	26%	19%
Relationship with colleagues	17%	12%	3%	11%
Job Function	9%	4%	1%	5%
Income	6%	2%	3%	4%

a Clusters with ≥40% agreement are in bold. ^b^OP  =  occupational physician. ^c^Average percentage is calculated as: (% employees + % supervisors + % OP’s)/3.

### Selection of Criteria (Table 3)

**Table 3:** Percentage of employees, supervisors, and occupational physicians (OP’s) who regarded a certain criterion most important to a successful RTW outcome.^a^

Sustainability (max. 1 criterion)	Employees	Supervisors	OP’s
	(n = 51)	(n = 51)	(n = 74)
A) Employee can function without relapse for a minimum period of:	31%	**61%**	**69%**
* 1–4 weeks*	*0%* ^ b^	*10%* ^ b^	*0%* ^ b^
* 2–3 months*	*6%* ^ b^	*12%* ^ b^	*16%* ^ b^
* 4–6 months*	*6%* ^ b^	*25%* ^ b^	*25%* ^ b^
* 7–12 months*	*44%* ^ b^	*42%* ^ b^	*25%* ^ b^
>*12 months*	*44%* ^ b^	*23%* ^ b^	*33%* ^ b^
B) Employee does not feel that he is bordering on relapse	**69%**	39%	31%
**At-work functioning (max. 3 criteria)^c^**	**Employees**	**Supervisors**	**OP’s**
	**(n = 52)**	**(n = 50)**	**(n = 74)**
A) Employee is able to complete his work within the allocated time period	14%	22%	27%
B) Employee is able to have functional relationships at work (e.g. show up formeetings)	25%	28%	16%
C) Employee is able to adequately cope with changes in the work environment	33%	24%	18%
D) Employee is able to think in a (sufficiently) problem solving manner	15%	16%	18%
E) Employee fulfils the tasks agreed upon with the employer	15%	**54%**	**46%**
F) Hours worked are economically productive	12%	24%	34%
G) Employee’s energy level is sufficient to fulfil work requirements	**71%**	**58%**	**69%**
H) Employee’s creativity level is sufficient to fulfil work requirements	6%	4%	8%
I) Employee’s concentration level is sufficient to fulfil work requirements	**69%**	**60%**	**49%**
J) Employee’s memory is sufficient to fulfil work requirements	25%	4%	12%
**Work-home balance (max. 2 criteria)^c^**	**Employees**	**Supervisors**	**OP’s**
	**(n = 53)**	**(n = 50)**	**(n = 72)**
A) Employee is able to pursue hobby’s	15%	2%	5%
B) Employee’s identity is no longer, for the most part, defined by work	30%	12%	18%
C) Situation at home does not suffer from the employee’s situation at work	**42%**	**54%**	38%
D) Situation at work does not suffer from the employee’s situation at home	25%	**66%**	**43%**
E) After a day at work, the employee has enough energy left for other activities	**76%**	**44%**	**71%**
**Mental functioning (max. 2 criteria)^c^**	**Employees**	**Supervisors**	**OP’s**
	**(n = 52)**	**(n = 50)**	**(n = 69)**
A) Employee has returned to work with only limited psychological symptoms	2%	13%	20%
B) The employee’s psychological well-being is comparable to that of healthy employees	35%	16%	16%
C) The morning after a workday, the employee wakes up rested	22%	4%	9%
D) The occupational physician estimates that the return to work does not posea threat for the employee’s recovery from his disorder	6%	**42%**	35%
E) Employee is able to recognize stress signals	**54%**	**43%**	38%
F) Employee has the insight and skills to deal with his psychological vulnerability	**73%**	**78%**	**71%**
**Job satisfaction (max. 2 criteria)^c^**	**Employees**	**Supervisors**	**OP’s**
	**(n = 52)**	**(n = 50)**	**(n = 71)**
A) Employee gets energy from his work	25%	16%	27%
B) Employee can enjoy his work again	39%	18%	18%
C) Employee is satisfied with his work situation	33%	**44%**	**45%**
D) Employee is once again proud of what he is doing at work	19%	18%	11%
E) Employee once again has the feeling that he is participating in society	**48%**	32%	**47%**
F) Employee is motivated at work	27%	**54%**	37%
**Relationship with supervisor (max. 2 criteria)^c^**	**Employees**	**Supervisors**	**OP’s**
	**(n = 52)**	**(n = 51)**	**(n = 72)**
A) Supervisor values the employee	17%	6%	10%
B) Supervisor is satisfied with the work that the employee delivers	17%	18%	38%
C) Supervisor is convinced that the employee has recovered	6%	4%	0%
D) Employee can openly communicate with his supervisor about his psychological problems	**58%**	**64%**	21%
E) Supervisor trusts that the employee is able to handle the work load	25%	**43%**	**63%**
F) Employee feels that he can trust his supervisor	**67%**	**48%**	**54%**
**Work load (max. 1 criterion)**	**Employees**	**Supervisors**	**OP’s**
	**(n = 48)**	**(n = 47)**	**(n = 71)**
A) Employee can do the full work load corresponding to his job function	15%	6%	10%
B) Workload is adjusted to the employee’s maximal capacity	5%	4%	14%
C) Employee and employer have achieved consensus regarding the job content/work load	**80%**	**90%**	**76%**
**Number of hours worked (max. 1 criterion)**	**Employees**	**Supervisors**	**OP’s**
	**(n = 49)**	**(n = 46)**	**(n = 73)**
Employee works a minimum of…. percent of:	62.3 (29.7)^d^	65.0 (29.5)^ d^	59.2 (29.1)^ d^
A) his current contract	20%	28%	26%
B) his old contract (before sickness absence)	25%	9%	16%
C) Employee and employer have achieved consensus regarding the number of hours worked	**55%**	**63%**	**58%**
**Relationship with colleagues (max. 2 criteria)^c^**	**Employees**	**Supervisors**	**OP’s**
	**(n = 53)**	**(n = 50)**	**(n = 72)**
A) Colleagues value the employee	23%	20%	18%
B) Employee is not afraid to ask help from colleagues	**62%**	**62%**	**51%**
C) Colleagues trust that the employee is able to handle the work load	**40%**	**54%**	**67%**
D) Employee feels he can trust his colleagues	**59%**	**52%**	**46%**
**Job Function (max. 1 criterion)**	**Employees**	**Supervisors**	**OP’s**
	**(n = 43)**	**(n = 40)**	**(n = 64)**
A) Employee has returned to a job function that is of an equivalent level as the job function before his sickness absence	21%	10%	20%
B) Employee has returned to work in a job that he experiences as suitable	30%	0%	0%
C) Employee has returned to work in a job that both the employee and supervisor experience as suitable	**49%**	**90%**	**80%**
**Income (max. 1 criterion)**	**Employees**	**Supervisors**	**OP’s**
	**(n = 45)**	**(n = 42)**	**(n = 68)**
A) Employee is no longer dependent on (government) benefits (WIA, WW, bijstand)^e^	18%	14%	23%
B) Employee feels that his income is sufficient	**41%**	21%	27%
C) Employee’s income corresponds with the job function in which the employee returns to	21%	**60%**	**44%**
D) Employee has just as much income as before he became sick	20%	5%	6%

a Only participants who rated a specific cluster as important in step 1 of the questionnaire are included in these analyses. ^b^ Percentages represent those participants who regarded criterion “A” as most important for successful RTW. ^c^ As participants were able to select more than one criterion, the percentages do not sum to a total of 100%. ^d^ Mean (SD). ^e^ WIA  =  disability pension; WW  =  unemployment benefits; bijstand  =  welfare.

#### Sustainability

While employees regarded their subjective experience (criterion B: ‘not having the feeling of bordering on relapse’) important for successful RTW, supervisors and OP’s found it important that the employee functions without relapse for a certain minimum period (criterion A). All three stakeholder groups agreed that at least seven months without relapse were required for successful RTW.

#### At-work functioning

All three stakeholder groups considered the employee’s energy level (criterion G) and the employee’s concentration level (criterion I) important for successful RTW. Supervisors and OP’s also found the extent to which the employee fulfils the agreements made with the supervisor (criterion E) important.

#### Work-Home balance

All three stakeholder groups considered the employee’s energy level after a working day (criterion E) important for successful RTW. In addition, employees and supervisors found it important that the employee’s home situation does not suffer from the work situation (criterion C), while OP’s and supervisors found it important that the employee’s work situation does not suffer from the home situation (criterion D).

#### Mental functioning

All three stakeholder groups found it important that an employee has insight and skills to deal with his psychological vulnerability (criterion F). Employees and supervisors regarded the employee’s ability to recognize stress signals (criterion E) also as an important criterion. Supervisors found it important that the OP estimates that the RTW does not pose a threat for the employee’s recovery (criterion D).

#### Job satisfaction

For successful RTW, supervisors and OP’s found it important that the employee is satisfied with his work situation (criterion C). Employees and OP’s found it important that the employee has the feeling that he is participating in society (criterion E); supervisors found it important that the employee is motivated at work (criterion F).

#### Relationship with supervisor

All three stakeholder groups regarded the employee’s trust in his supervisor (criterion F) important for successful RTW. Employees and supervisors agreed that open communication between the supervisor and employee about the psychological problems (criterion D) was an important criterion. Supervisors and OP’s found it important that the supervisor trusts that the employee is able to handle the work load (criterion E).

#### Work load

All three stakeholder groups considered consensus between the employee and supervisor regarding the work load (criterion C) an important criterion for successful RTW.

#### Number of hours worked

All three stakeholder groups selected the consensus between the employee and supervisor regarding the number of hours worked (criterion C) as an important criterion. With respect to the minimum percentage of hours worked for evaluating a RTW as successful, all three stakeholder groups required at least 60% of the total number of contract hours. Thus, stakeholders did not necessarily consider a full RTW needed in order to evaluate a RTW as successful.

#### Relationship with colleagues

All three stakeholder groups considered criterion B (employee is not afraid to ask help from colleagues), criterion C (colleagues trust that the employee is able to handle the work load), and criterion D (employee feels he can trust his colleagues) important criteria for successful RTW.

#### Job function

All three stakeholder groups found it important that the employee has returned to work in a job that both the employee and supervisor experience as suitable (criterion C).

#### Income

Whereas employees considered the feeling that their income is sufficient (criterion B) important for successful RTW, supervisors and OP’s found it important that the employee’s income corresponds with the job function to which the employee returns (criterion C).

## Discussion

The present study found that key stakeholders vary in the aspects they regard as important to successful RTW after CMD-related sickness absence. Although all three stakeholder groups selected sustainability to be important, supervisors and OP’s more frequently regarded at-work functioning important, while employees more frequently considered job satisfaction, work-home balance, and mental functioning important. Similarly, we found that key stakeholder perspectives may differ regarding the importance of specific criteria for successful RTW: For example, despite agreement regarding the importance of sustainability, employees regarded their subjective experience as more important for successful RTW, whereas supervisors and OP’s regarded the minimum period of functioning without relapse as more important. Finally, we found considerable heterogeneity in perspectives, not only *between* stakeholder groups, but also *within* stakeholder groups. Most clusters and criteria identified as important by a specific stakeholder group did not exceed an agreement of 55–60%.

These findings have important implications for current definitions of RTW outcomes in scientific research: First, no single definition may be sufficient to adequately reflect the complex reality underlying successful RTW. Instead, different outcomes may be considered ‘successful’, depending on the stakeholder perspective. Thus, conclusions concerning the effectiveness of an intervention in achieving successful RTW will depend on the *perspective* from which these outcomes are evaluated.

Second, traditional research definitions of RTW outcomes (e.g. hours worked, income earned) may not adequately capture those aspects most important to the key stakeholders. Only a few studies have included at-work productivity [4], [6] or relapse [4], [7] when evaluating the effectiveness of RTW interventions after CMD-related sickness absence. This is particularly surprising given that CMD’s are often characterized by a recurrent nature [31] and that the majority of work-related costs are related to reduced at-work productivity [32]. Even when a sustainability criterion is included (e.g. 4 weeks) [6], [9], all three stakeholder groups regarded a much longer minimum period required (at least 7 months) to evaluate a RTW as successful. Furthermore, most aspects identified as important by employees (e.g. job satisfaction, home-work interference) have, to our knowledge, not yet been included in current RTW definitions [7].

Interestingly, most criteria traditionally used for defining RTW outcomes (e.g. hours worked, income earned) were among the *least* important aspects from the key stakeholders’ perspectives. All three stakeholder groups did not necessarily consider full RTW a prerequisite for successful RTW, but instead regarded a ‘subjective’ criterion (i.e., consensus between supervisor and worker) more important for successful RTW. These results are corroborated by previous findings that partial RTW may be a long-term solution for some workers with reduced work ability [33]. In addition, all three stakeholder groups considered the traditional criterion of equal earnings as before sickness absence *least* important for successful RTW: Instead, most important to employees was their feeling that their income is sufficient, whereas supervisors and OP’s found it most important that the employee’s income level corresponds to the job function to which the employee returns.

### Strengths and Limitations

This study has several strengths: the examination of different stakeholder perspectives, a large sample size, and the use of both qualitative and quantitative methods. A study limitation may be the generalizability of our findings. Most participants in study phase 2 were from large companies: As larger workplaces often have more opportunities to accommodate changes in job function and work load, these aspects may have been rated less important when compared to smaller workplaces. In addition, most participants were recruited from the financial or healthcare sector, which may have also affected the generalizability of our study results.

However, exploratory analyses stratified for company size (<150 employees vs. >150 employees), sector (financial, healthcare, other), and other relevant baseline characteristics (i.e., diagnosis, current percentage of work resumption) revealed that even within subgroups, traditional research criteria for defining RTW (i.e. hours worked, income earned, job function) were considered *least* important for all three stakeholder groups (data available from first author). This supports our study conclusion that traditional research definitions do not accurately reflect key stakeholder perspectives. Furthermore, despite the fact that most participants were from the financial/healthcare sector and from larger companies, our results still show considerable heterogeneity in perspectives. It is likely that these differences are even more pronounced in a more heterogeneous sample.

Finally, it is important to note the legislative context in which this research has been conducted. In the Netherlands, the employer is financially responsible for the worker’s salary during the first two years of sickness absence. During this period, both the employer and worker are legally obligated to maximize their effort into having the worker RTW, either in the former or a new work situation. Other countries with different insurance structures and compensation schemes may find different results. Our findings should therefore be replicated in smaller companies, other sectors, and other jurisdictions.

### Conclusions

To our knowledge, this is the first study to empirically examine the definition of successful RTW after CMD-related sickness absence from a multi-stakeholder perspective. Our findings underline the importance of taking into account the *perspective* from which to evaluate a successful RTW, as no single definition may adequately reflect the complex reality underlying this concept. Considering that successful RTW is not necessarily disease-specific, our findings could also be useful for defining successful RTW after common physical conditions, such as low back pain. However, future studies should examine to what extent our findings are generalizable to other health conditions.

Considering the marked increase in CMD-related sickness absence, there is an urgent need to further clarify these different perspectives on successful RTW, including those of health insurers and mental health professionals. More knowledge regarding these different perspectives may not only promote the evaluation, but also the development and implementation of new RTW interventions.
